# 

**Published:** 1885-04

**Authors:** 


### BOOKS RECEIVED.

---

*Fifth Annual Report of the State Board of Health, Lunacy and Charity, of Massachusetts.* Boston. 1884.

*A Text-Book of Hygiene,* by Geo. H. Rohe, M. D. Thomas & Evans, Baltimore.

*A Manual of Dissection of the Human Body,* by Luther Holden. Philadelphia: P. Blakiston, Son & Co.

*The Diagnosis and Treatment of Chronic Nasal Catarrh.* Three clinical lectures delivered at the College of Physicians and Surgeons, New York, by George Morewood Lefferts, A. M., M. D. St. Louis: Lambert & Co.

*Diseases of the Urinary Male Sexual Organs,* by William T. Belfield, M. D. October Number Wood's Library of Standard Medical Authors. William Wood & Co., N. Y.

*A Manual of the Medical Botany of North America,* by Lawrence Johnson, A. M., M. D. New York: William Wood & Co. December Number Wood's Library of Standard Medical Authors.

*The Therapeutics of the Respiratory Passages,* by Prosser James, M. D. November Number of Wood's Library of Standard Medical Authors. William Wood & Co., N. Y.

*The International Encyclopædia of Surgery.* Vol. V. William Wood & Co., N. Y.

*A System of Practical Medicine by American Authors*, edited by Wm. Pepper. Vol. V. Pathology and General Diseases. Lea Bros. & Co., Philadelphia, Pa.

*Consumption: Its Nature, Causes, Prevention and Cure*, by Jno. Kitchen, M. D. New York: G. P. Putnam's Sons.

*Human Osteology*, by Luther Holden. Wood's Library of Standard Medical Authors. January, 1885.

(BOUND.)

*Practical Anatomy: A Manual of Dissections*, by Christopher Heath, F. R. C. S. Philadelphia: P. Blakiston, Son & Co.

*Handbook of the Diagnosis and Treatment of Skin Diseases*, by Arthur Van Harlingen. Philadelphia: P. Blakiston, Son & Co.

*A Handbook of Pathological Anatomy and Histology, with an Introductory Section on Post-Mortem Examinations and the Methods of Preserving and Examining Diseased Tissues*, by Francis Delafield, M. D., and T. Mitchell Prudden, M. D. New York: Wm. Wood & Co. Chicago: W. T. Keener.

*Kirke's Handbook of Physiology*, by W. Morrant Baker and Vincent Dormer Harris. 11th Edition. New York: Wm. Wood & Co. Chicago: W. T. Keener.

*The Year Book of Treatment for 1884.* A critical review for practitioners of medicine and surgery. Philadelphia: Lea Bros. & Co. Chicago: S. A. Maxwell & Co.

*Saint Bartholomew's Hospital Reports*, edited by W. S. Church, M. D., and John Langton, F. R. C. S. London: Smith, Elder & Co.

*De L'Aphosie et de ses Diverses Formes*, par de Dr. Bernard. Publications du Progrés Medical.

*Transactions of the American Surgical Association.* Vol. II. Edited by J. Ewing Mears. Philadelphia: P. Blakiston, Son & Co.

*The Student's Manual of Histology*, by Chas. H. Stowell, M. D. Third Edition. Ann Arbor.

*The Physician Himself*, by D. W. Cathell, M. D. Fourth Edition. Baltimore: Cushing & Bailey.

*Insomnia and Other Disorders of Sleep*, by Henry M. Lyman, A. M., M. D.. Chicago: W. T. Keener.

*The London Medical Student and Other Comicalities*, selected and compiled by Hugo Ericham, M. D. Detroit: Free Press Printing Co.

*Lectures on Diseases of the Nervous System, especially in Women*, by S. Weir Mitchell, M. D. Lea Bros. & Co. Chicago: Jansen, McClurg & Co.

*A Manual of Organic Materia Medica*, by John M. Maisch, Phar. D. Lea Bros. & Co. Chicago: W. T. Keener.

*A Manual for the Practice of Surgery*, by Thomas Bryant, F. R. C. S. Henry C. Lea's Son & Co. Chicago: W. T. Keener.

*The Science and Art of Surgery*, by Jno. Eric Erichsen, F. R. S., LL. D., F. R. C. S. 8th Edition. Lea Bros. & Co. Chicago: Jansen, McClurg & Co.

*The Analectic*, edited by Walter S. Wells, M. D. G. P. Putnam's Sons. Chicago: S. A. Maxwell & Co.

*On Wasting Diseases of Infants and Children*, by Eustace Smith, M. D., London. 4th Edition. Wm. Wood & Co. Chicago: W. T. Keener.

*Transactions of the 34th Annual Meeting of the Illinois State Medical Society*. 1884.

*Elements of Practical Medicine*, by Alfred H. Carter, M. D., London. D. Appleton & Co. Chicago: Jansen, McClurg & Co.

*Elements of Surgical Diognosis*, by A. Pearce Gould, M. S., M. B., Lond., F. R. C. S., Eng. Henry C. Lea's Son & Co. Chicago: Jansen, McClurg & Co.

*Insanity and Allied Neuroses*, by Geo. H. Savage, M. D., M. R. C. P. Henry C. Lea's Son & Co. Chicago: Jansen, McClurg & Co.

*Intestinal Obstruction*, by Frederick Treres, F. R. C. S. Henry C. Lea's Son & Co. Chicago: W. T. Keener.

*A Guide to the Diseases of Children*, by Jos. Frederick Goodhart, M. D., F. R. C. P. P. Blakiston, Son & Co. Chicago: W. T. Keener.

*Guy's Hospital Reports*. Vol. XLII.

*One Hundred Years of Publishing*. Lea Bros. & Co.

*Cocaine and its Use in Ophthalmic and General Surgery*, by H. Knapp, M. D. G. P. Putnam's Sons. Chicago: S. A. Maxwell & Co.

*A Treatise on Amputation of the Extremities and their Complications*, by R. A. Watson, A. M., M. D. P. Blakiston, Son & Co. Chicago: W. T. Keener.

### PAMPHLETS RECEIVED.

Memoir on the Nature of Diphtheria. By Drs. H. C. Wood and H. F. Formad.

The Curability of Locomotor Ataxia and the Simulations of Posterior Spinal Sclerosis. By C. H. Hughes, M. D.

Gunshot Wounds of the Small Intestines. By Charles T. Parkes, M. D.

Poisoning by Cannabis Indica. A. B. Cook, M. D.

Premiére Application a Paris en 1883. L'Assainis sement suirant le Systéme Waring par Ernest Pontzen Ingèniem Ciril.

Preventable Blindness. Samuel Theobald, M. D.

Ziemssen's Motor Points of the Human Body—A Guide to Localized Electrization. Herbert Tebbetts, M. D.

Proceedings and Addresses at a Sanitary Convention. held at Ionia, Mich., Dec. 13 and 14, 1883.

Laws of Michigan Relating to Public Health. In force Sept. 8, 1883.

Transactions of the Medical Society of West Virginia, 1884.

Transactions of the Medical and Chirurgical Faculty of Maryland. 86th Annual Session.

Cases of Reflex Cough due to Nasal Polypi: with Remarks. Jno. N. Mackenzie.

Transactions of the American Otological Society—17th Annual Meeting.

Kefyr Kaukasisches Gährungs ferment und Getröuk ans Kuhmilch. Dr. W. Podwyssotzki.

Explanation of the Pathology and Therapeutics of the Nervous System: Especially Epilepsy. J. McF. Gaston, M. D.

Contributions to the Anatomy and Pathology of the Nervous System. Singular Case of Vertebral Disease. Richard Mollenhauer.

Mumps as a Cause of Sudden Deafness. Leartus Connor, M. D.

A Clinical Study of Syphilis of the Eye and its Appendages. Leartus Connor, M. D.

Notes on the Treatment of Trochoma by Jequirity. Leartus Connor, M. D.

Club Foot. Is Excision of the Sorsus Necessary in Children? DeF. Willard, M. D.

Experimentelle Untersuchuagen und Studien siber Contracturen der Stinunbandmuskeln. Von Dr. H. Krause in Berlin.

The Relation of Micro-Organisms to Surgical Lesions. Henry O. Marcy, M. D.

On Branchial Cysts of the Neck. By N. Senn, M. D.

Official Register of Physicians and Midwives (now in practice) to whom Certificates have been issued by the State Board of Health of Illinois. 1884.

Transactions of the Michigan State Medical Society for the year 1884.

The Plaster of Paris Dressing in the Treatment of Fractures. W. O. Daniel, M. D.

A Contribution to the Relations of Orulation and Menstruation. A. Reeves Jackson.

On Oxygen as a Remedial Agent. S. S. Wallian, M. D.

Conspectus of the Medical Colleges of America. Sessions of 1884–5. Illinois State Board of Health.

The Rôle of Bacteria in Infectious Diseases. Henry O. Marcy.

A Useful Catheter for the Operation for Vesico-Vaginal Fistula.

School Hygiene in Relation to its Influence upon the Vision of Children, or School Sanitation. A. W. Calhoun, M. D.

Acetate of Lead in Ocular Therapeutics. David DeBeck, M. D.

Des Arantages de L'Hydrotherapie Hirernale. par E. Duval.

Typhoid Fever and Low Water in Wells. Henry B. Baker, M. D.

Proceedings of the Philadelphia Co. Medical Society. Vol. VI.

Medical Jurisprudence in Divorce. Carl H. Von Klein, A. M., M. D.

Experimental Researches on Cicatrization in Blood Vessels after Ligature. N. Senn, M. D.

Seventh Annual Report of the Presbyterian Eye, Ear and Throat Charity Hospital. Baltimore. 1885.

Remarks on Typhoid Fever in the Young. A. Jacobi, M. D.

Inaugural Address delivered before the New York Academy of Medicine. A. Jacobi, M. D.

Catalepsy in a Child Three Years Old. A. Jacobi, M. D.

---

AMERICAN MEDICAL ASSOCIATION.—The thirty-sixth annual session of the American Medical Association will be held in New Orleans, La., on Tuesday, Wednesday, Thursday and Friday, April 28th, 29th, 30th and May 1st, 1885, commencing on Tuesday, at 11 A. M.

Dr. James Lawrence Little, Professor of Clinical and Operative Surgery in the New York Post-Graduate Medical School, died suddenly in New York, April 4th, at the age of 49.

Dr. Abner Hard, of Aurora, died in that city on March 20th, from blood-poisoning.

SUBSCRIPTION PRICE, $3.00 PER YEAR.

Whole Number, 304. APRIL, 1885. Vol. L., Number 4.

LIBRARY
INDEXED
S. G. O.

### THE CHICAGO MEDICAL JOURNAL AND EXAMINER

EDITORS:

S. J. JONES, M.D., LL.D.

W. W. JAGGARD, A.M., M.D.

HAROLD N. MOYER, M.D.

COLLABORATORS.

CHICAGO:

PROFESSOR J. A. ALLEN, M. D., LL. D.

PROFESSOR E. ANDREWS, M. D., LL. D.

PROFESSOR W. H. BYFORD, A. M., M. D.

PROFESSOR O. C. DEWOLF, A. M., M. D.

PROFESSOR E. C. DUDLEY, A. B., M. D.

PROFESSOR J. H. ETHERIDGE, A. M., M. D.

PROFESSOR C. FENGER, M. D.

PROFESSOR J. N. HYDE, A. M., M. D.

PROFESSOR H. A. JOHNSON, M. D., LL. D.

CHARLES GILMAN SMITH, A. M., M.D.

J. F. TODD, M. D.

MILWAUKEE, WISCONSIN:
N. SENN, M, D.

WASHINGTON, D. C:
S. O. RICHEY, M. D.

UNITED STATES NAVY:
MEDICAL DIRECTOR J. M. BROWNE, M. D.

CHICAGO:

